# Identification and Molecular Characterization of Microneme 5 of *Eimeria acervulina*

**DOI:** 10.1371/journal.pone.0115411

**Published:** 2014-12-22

**Authors:** ZhenChao Zhang, JingWei Huang, MengHui Li, YuXia Sui, Shuai Wang, LianRui Liu, LiXin Xu, RuoFeng Yan, XiaoKai Song, XiangRui Li

**Affiliations:** College of Veterinary Medicine, Nanjing Agricultural University, Nanjing, Jiangsu, PR China; Instituto Butantan, Brazil

## Abstract

In the present study, the microneme 5 gene of *Eimeria acervulina* (*E. acervulina*) (EaMIC5) was cloned and characterized. Specific primers for the rapid amplification of cDNA ends (RACE) were designed based on the expressed sequence tag (EST, GenBank Accession No. EH386430.1) to amplify the 3′- and 5′-ends of EaMIC5. The full length cDNA of this gene was obtained by overlapping the sequences of 3′- and 5′-extremities and amplification by reverse transcription PCR. Sequence analysis revealed that the open reading frame (ORF) of EaMIC5 was 336 bp and encoded a protein of 111 amino acids with 12.18 kDa. The ORF was inserted into pET-32a (+) to produce recombinant EaMIC5. Using western blotting assay, the recombinant protein was successfully recognized by the sera of chicks experimentally infected with *E. acervulina*, while the native protein in the somatic extract of sporozoites was as well detected by sera from rats immunized with the recombinant protein of EaMIC5. Immunofluorescence analysis using antibody against recombinant protein EaMIC5 indicated that this protein was expressed in the sporozoites and merozoites stages of *E. acervulina*. Animal challenge experiments demonstrated that the recombinant protein of EaMIC5 could significantly increase the average body weight gains, decrease the mean lesion scores and the oocyst outputs of the immunized chickens, and presented anti-coccidial index (ACI) more than 160. All the above results suggested that the EaMIC5 was a novel *E. acervulina* antigen and could be an effective candidate for the development of a new vaccine against this parasite.

## Introduction

*E. acervulina* is one of the seven *Eimeria* spp which infect the chicken. This parasite infects the intestinal epithelial cells of chicken and can cause intense damage to duodenum, result in malabsorption and poor feed utilization, and reduced body weight gain [1]–[3]. Chicken coccidiosis exists worldwide and is economically the most important parasitic disease of the poultry industry. So far, the main measures of controlling *Eimeria* infection are applications of anti-coccidial drugs and live vaccines. However, the emergence of drug resistance, the potential reversion to virulence and high production expenses of live vaccines have driven the developments of new control strategies [4].

Recent efforts are committed to find recombinant antigen or DNA vaccines against coccidiosis [5]–[7]. Some studies have proved that the recombinant antigen or DNA vaccines can induce both humoral and cell-mediated immune responses [8]–[10]. Meanwhile, cytokines as adjuvants have been considered to enhance the potential of DNA vaccines or recombinant antigen to induce broad and long-lasting humoral and cellular immunity [11], [12].

Microneme organelles are present in all apicomplexan protozoa and contain proteins critical and multifunctional for parasite motility and host cell invasion [13]. So far, nine microneme proteins have been reported in *Eimeria*. They are microneme protein 1–7 (MIC1-7) and apical membrane antigen 1, 2 (AMA1,2) [14]. The functions of MICs during host cell invasion suggest they might be potential candidates for vaccines development against the infection of *Eimeria*. The gene sequences of *E. tenella* MIC1 (AF032905.1), MIC2 (KC333870.1), MIC3 (AY512382.1), MIC4 (AJ306453.2), MIC5 (AJ245536.1) and AMA1 (JN032081.1), *E. maxima* MIC2 (FR718971.1), MIC3 (FR718972.1), MIC5 (FR718974.1) and MIC7 (FR718975.1) and *E. necatrix* MIC5 (EU335049.1) were published in GenBank.

The EtMIC5 is a micronemal glycoprotein and has eleven cysteine-rich receptor-like regions with striking similarity to the Apple domains (A-domains) of the binding regions of blood coagulation factor XI (FXI) [15] and plasma pre-kallikrein (PK) [16]. When sporozoites were in contact with host cell, EtMIC5 was secreted by the sporozoite [17]. Saouros et al [18] demonstrated the C-terminal region of TgMIC5, the MIC5 of *Toxoplasma gondii*, was responsible for inhibition of TgSUB1, while Periz et al [19] suggested EtMIC4 and EtMIC5 formed an oligomeric, ultrahigh molecular mass protein complex that interacted with target host cells during invasion.

Until now, few genes of *E. acervulina* have been reported and tested for their immunogenicity, and no MIC of it is reported and characterized although there is EST in GenBank.

In this study, the gene of EaMIC5 was obtained, characterized and the immunogenicity of the recombinant protein of EaMIC5 was checked through chicken challenge experiments.

## Materials and Methods

### Animals and parasites

New-hatched Chinese Yellow chickens were reared in clean brooder cages under coccidian-free conditions and were screened periodically for their *Eimeria* infection status by microscopic examination of feces. The birds were provided with coccidiostat-free feed and water ad libitum. The birds were shifted to animal containment facility prior to challenge with virulent oocysts. The study was conducted following the guidelines of the Animal Ethics Committee, Nanjing Agricultural University, China. All experimental protocols were approved by the Science and Technology Agency of Jiangsu Province. The approval ID is SYXK (SU) 2010-0005.

*E. acervulina* JS strain was propagated and maintained in the Laboratory of Veterinary Parasite Disease, Nanjing Agricultural University, China. Sporulated oocysts of *E. acervulina* JS strain were stored in 2.5% potassium dichromate solution at 4°C and passed through chickens every 5 months interval.

Sporozoites from *E. acervulina* oocysts were purified on DE-52 anion-exchange columns using a protocol described previously [20]. *E. acervulina* merozoites were harvested from the duodenal loops of chickens 54 h post-infection (p.i.) and purified using standard methods [21], [22] before being pelleted and frozen in liquid nitrogen.

### Soluble antigens of *E. acervulina*

A total of 5×10^9^ *E. acervulina* sporozoites were washed three times by centrifugation with 0.1 M PBS (pH 7.2) at 2000×g for 10 min at 4°C. The pellet was dissolved respectively in 2 ml of PBS and PBS containing 0.5% TritonX-100 and was disrupted by ultrasound in ice bath (200 W, work time 5 s, interval time 10 s, 50 cycles). After high-speed centrifugation, the supernatant proteins were separated and estimated spectrophotometrically, adjusted to 1 mg/ml with PBS and stored at −20°C until to be used.

The soluble antigen dissolved by PBS containing Triton X-100 was used for western blot to analyze the native protein of the EaMIC5.

### Cloning of EaMIC5 gene

#### RNA extraction

Total RNA was extracted from *E. acervulina* sporozoites using TRIZOL reagent (TaKaRa) according to the manufacture's instructions. RNA samples were resuspended in diethyl pyrocarbonate (DEPC) treated water in the presence of ribonuclease inhibitor (TaKaRa). All RNA samples were treated with RNase-free DNase I (TaKaRa) before processing reverse transcription to eliminate genomic DNA contamination. The quantity of RNA was estimated by measuring the optical density at 260 nm (OD_260_) using a spectrophotometry and the quality was determined by OD_260_/OD_280_ ratio. The samples with ratio OD_260_/OD_280_ between 1.9 and 2 were used.

#### 3′- and 5′-rapid amplification of cDNA ends

A 3′-end of the cDNA was amplified by 3′-full RACE kit (TaKaRa Biotech, Dalian, PR China) using the forward gene specific primers EaMIC5-3-F1 and EaMIC5-3-F2 (Table 1) designed based on EaMIC5 EST (GenBank Accession No. EH386430.1) in combination with the 3′outer and 3′inner primers provided in the RACE kit (Table 1). The primary PCR system and condition were set as the manufacturer's protocol described. The EaMIC5 3′-end fragment was then obtained and sequenced.

**Table 1:** Oligonucleotide primer sequences used for PCR in this research.

Name	Sequences (5′→3′)	Description
EaMIC5-3-F1	ATCCAGATACTTTGCCTTGTTTTT	Forward primer specific for 3′-end of EaMIC5 in primary PCR
EaMIC5-3-F2	ACATCGGGTGTTTGTAGCAGA	Forward primer specific for 3′-end of EaMIC5 in second PCR
3′outer primer	TACCGTCGTTCCACTAGTGATTT	Reverse primer for 3′-end of EaMIC5 in primary PCR (in RACE kit)
3′inner primer	CGCGGATCCTCCACTAGTGATTTCACTATAGG	Reverse primer for 3′-end ofEaMIC5 in second PCR (in RACE kit)
EaMIC5-5-R1	AACCTCCTTCAAGACTATTCCG	Reverse primer specific for 5′-end of EaMIC5 in primary PCR
EaMIC5-5-R2	ATGCGAATACGAGAGCAACTG	Reverse primer specific for 5′-end of EaMIC5 in second PCR
5′outer primer	CATGGCTACATGCTGACAGCCTA	Forward primer for 5′-end of EaMIC5 in primary PCR (in RACE kit)
5′inner primer	CGCGGATCCACAGCCTACTGATGATCAGTCGATG	Forward primer for 5′-end of EaMIC5 in second PCR (in RACE kit)
EaMIC5F	CCGGAATTC GAGCTCTGCTACAAACACCCG	Forward primer containing an *Eco*RI site for cloning into pET32a-EaMIC5
EaMIC5R	CCCAAGCTTGCCGTTATTAAATGCATGCG	Reverse primer containing a *Hin*dIII site for cloning into pET32a-EaMIC5

The 5′-end of the cDNA was amplified by 5′-RACE PCR using the same method as the 3′-RACE PCR. The primary PCR was performed using EaMIC5-5-R1 and 5′ outer primer, with EaMIC5-5-R2 and 5′ inner primer in the Nest PCR.

Both of the products of the second PCRs for 3′-end and 5′-end were cloned into the pMD18-T vector (TaKaRa Biotech, Dalian, PR China) and sequenced by Invitrogen Biotech (Shanghai, PR China).

All oligonucleotides used in this research were synthesized by Invitrogen Biotechnology Co. Ltd. (Shanghai, PR China).

### The amplification of the EaMIC5 ORF

The complete sequence of the EaMIC5 cDNA was deduced from the overlapping sequences of both 3′-end and 5′-end amplification products using BioEdit Version 7.0.1 (T.A. Hall, North Carolina State University, USA). Open Reading Frame Finder (ORF Finder) was used to predict the ORF of MIC5. The ORF was amplified from cDNA of sporozoites by PCR with the following restriction enzyme-anchored (underlined) and protective bases-anchored primers (*Eco*RI anchored forward primer, 5′-CCGGAATTCGAGCTCTGCTACAAACACCCG-3′, *Hin*dIII anchored reverse primer, 5′-CCCAAGCTTGCCGTTATTAAATGCATGCG-3′). PCR products were cloned into pMD18-T vector (TaKaRa Biotech, Dalian, PR China) and transformed into *E. coli* (DH5a) competent cells (Invitrogen). Recombinant pMD18-T-MIC5 clone was identified by PCR amplification and endonuclease digestion. Three positive clones were further confirmed by sequence analysis. The complete nucleotide sequence data inserted in recombinant plasmid was analyzed for homology to known sequences in GenBank databases using a basic alignment search tool (BLAST) (http://www.ncbi.nlm.nih.gov/BLAST/).

All oligonucleotides used in this research were synthesized by Invitrogen Biotechnology Co. Ltd. (Shanghai, PR China).

### Sequence analysis

Sequence similarity was studied using the BLASTP and BLASTX (http://www.blast.ncbi.nlm.nih.gov/Blast.cgi). Microneme protein sequences were aligned using CLUSTALW1.8. The signal peptide, secondary structure and protein motifs were predicted using approaches accessible on the Internet: SignalP (http://www.cbs.dtu.dk/services/SignalP/), TMHMM (http://www.cbs.dtu.dk/services/TMHMM/), GPI Modification Site Prediction (http://mendel.imp.ac.at/sat/gpi/gpi_server.html), PSIpred (http://www.bioinf4.cs.ucl.ac.uk:3000/psipred/), Motifscan (http://www.myhits.isb-sib.ch/cgibin/motif_scan), respectively.

### Expression and purification of MIC5 recombinant protein and pET-32a protein

The identified recombinant plasmid pMD18-T-MIC5 was digested with *Eco*R I and *Hin*dIII. The target fragment of MIC5 was purified and cloned into frame of digested expression vector pET-32a (+) (Novagen, USA) to generate plasmid pET-32a-MIC5. The recombinant plasmid was sequenced to confirm that the MIC5 insert was in the proper reading frame. The correct recombinant pET-32a-MIC5 plasmid was transferred into competent *E. coli* BL21 (DE3) cells and the recombinant protein expression in *E.coli* was induced by addition of 0.8 mM Isopropyl-β-D-thiogalactopyranoside (IPTG;Sigma–Aldrich, USA) to the cell culture after the OD_600_ of the culture reached 0.6 at 37°C. The cells were incubated at 37°C for 5 h and harvested by centrifugation after the addition of IPTG. The cell pellet was lysed using lysozyme (10 µg/ml) (Sigma–Aldrich, USA) followed by sonication and then the cell lysates were analyzed by 12% (w/v) sodium dodecyl sulfate polyacrylamide gel electrophoresis (SDS-PAGE).

The recombinant protein was purified by Ni^2+^-nitrilotriacetic acid (Ni-NTA) column (GE Healthcare, USA) according to the manufacturer's instructions. Purity of the protein was detected by 12% SDS-PAGE and refolded by renaturation buffer (20 mmol/L Tris-Cl,500 mmol/L NaCl,1 mmol/L GSH,0.1 mmol/L GSSG,pH 8.0) containing different concentrations of urea (8, 6, 4, 2, 0 mol/L). The concentration of refolded protein was determined according to the Bradford procedure [23], using bovine serum albumin (BSA) as a standard. The protein was stored at −20°C for later use.

The pET-32a protein was obtained by induction of *E. coli* BL21 transformed with pET-32a (+) plasmid using the same methods to the recombinant protein.

### Antisera against recombinant MIC5 protein and against *E. acervulina*

To generate polyclonal antibodies, about 0.3 mg of the purified recombinant MIC5 protein were mixed with Freund's complete adjuvant as a 1∶1 mixture and injected into SD rats (Qualitied Certificate: SCXK 2008-0004; Experimental Animal Center of Jiangsu, PR China) subcutaneously in multiple places. Two weeks later, a booster was given in the same conditions by using protein which was mixed with Freund's incomplete adjuvant as a 1∶1 mixture. And then, the rats were re-boosted three times at intervals of 1 week. Finally, the serum was collected and stored until used. Sera collected before protein injection were used as negative sera [24].

The antisera against *E. acervulina* (chicken antisera) were collected from chickens experimentally infected with *E. acervulina* 1 week post-infection.

### Immunoblotting analysis for the recombinant and native EaMIC5

Samples including crude somatic extracts of *E. acervulina* sporozoites and the recombinant protein of MIC5 were separated by SDS-PAGE. Then the proteins were transferred to nitrocellulose membrane (Millipore, USA). After being blocked with 5% (w/v) skimmed milk powder in TBS (Tris-buffer saline)–Tween 20 (TBST), the membranes were incubated with the primary antibody (rat antisera and chicken antisera, respectively) for 1 h at 37°C (dilutions 1∶100 to rat antisera, 1∶100 to chicken antisera). Then the membranes were washed 3 times and were incubated with horseradish peroxidase (HRP)-conjugated goat anti-rat IgG and HRP-conjugated donkey anti-chick IgG (Sigma, USA) at 37°C for 1 h, respectively. Finally, the bound antibody was detected using 3,3′-diaminobenzidine tetrahydrochloride (DAB) kit (Boster Bio-technology) according to the manufacturer's instructions.

### Expression analysis of EaMIC5 in sporozoite and merozoite of *E. acervulina* by immunofluorescence

Purified sporozoites and merozoites were smeared and air-dried on a poly-L-lysine treated glass slide before fixation. Sporozoites and merozoites were fixed with 4% paraformaldehyde in TBS for 10 min at RT, permeabilized with 1% TritonX-100 in TBS for 10 min, washed 3 times in TBS containing 0.05% Tween-20 (TBST), and blocked with TBST containing 5% (w/v) BSA for 2 h at 37°C. After 3 times washing in TBST, rat antiserums against EaMIC5 (1∶100 dilution) were added respectively and allowed to incubate at 4°C overnight. After 3 washes in TBST, the coverslips were maintained in darkness for 40 min in goat anti-rat IgG antibody (Beyotime) labeled with Cy3 diluted at 1∶1000. After washing with TBST, fluorescent mounting medium (Beyotime) was added and cells were examined by fluorescent microscopy (Olympus).

### Immunization and challenge infection

Two-week-old chickens were randomly divided into five groups of 30 each as shown in Table 2 according to the permission for inoculating the chickens with the recombinant protein vaccine issued by the Animal Care and Ethics Committee of Nanjing Agricultural University. Experimental group was inoculated with 200 µg EaMIC5 recombinant protein without any adjuvant, the pET-32a protein control group was given 200 µg of pET-32a protein without adjuvant, and the PBS control group was given the same volume of PBS alone each chicken at the same injection site. The chickens of challenged control and unchallenged control groups were not injected. A booster immunization was given 1 week later with the same amount of components as the first immunization. Seven days post second injection, the chickens in each group except the unchallenged control group were challenged orally with 1.2×10^5^ sporulated oocysts of *E. acervulina*. Unchallenged control chickens were given PBS orally. All of the chickens were euthanized following protocols approved by the Animal Care and Ethics Committee of Nanjing Agricultural University to determine the effects of immunization on the 6th day post-challenge.

**Table 2:** Effects of recombinant EaMIC5 protein against *E. acervulina* challenge on different parameters.

Groups	Average body weight gains (g)	Mean lesion scores (difference in rank sum)	Oocyst out (lg) (mean±SD)put	Oocyst decrease ratio(%)	Anti-coccidial index
Unchallenged control	140.07±1.27^a^	0^a^	—^a^	100^a^	200
pET-32a protein control	68.95±4.51^c^	98.57^c^	5.43±0.02^c^	1.26^c^	82.64
PBS control	68.24±4.61^c^	101.8^c^	5.43±0.02^c^	1.50^c^	81.03
Recombinant MIC5 protein	121.55±2.81^b^	49.23^b^	4.88±0.03^b^	72.26^b^	161.24
Challenged control	67.27±4.37^c^	104.4^c^	5.44±0.03^c^	0.00^c^	79.86

Note: in each column, significant difference (p<0.05) between means and ranks with different letters and no significant difference (p>0.05) between means and ranks with the same letter; the oocyst output was zero (designated as “—”) in the unchallenged control group.

### Evaluation of immune protection

The efficacy of immunization was evaluated on the basis of lesion score, body weight gain, oocyst output, oocyst decrease ratio and anti-coccidial index (ACI) [25], [26]. Body weight gain of chickens in each group was determined by weighing the chickens at the end of the experiments subtracting the body weight at the time of challenge. Lesion scores were observed and recorded according to the system described by Reid and Johnson [27]. The duodenal content for each group was collected separately and oocysts per gram of content (OPG) were determined using McMaster's counting technique. Oocyst decrease ratio was calculated as described by Rose and Mockett [28] and Talebi and Mulcahy [29] as follows: (the number of oocysts from the challenged control chickens − vaccinated chickens)/the challenged control chickens×100%.

ACI was a synthetic criterion for assessing the protective effect of a medicine or vaccine and calculated as follows: (survival rate + relative rate of weight gain) − (lesion value + oocyst value). Whereas, survival rate was estimated by the number of surviving chickens divided by the number of initial chicken. Relative rate of weight gain of the chickens in each group was determined by subtracting the body weight of the chickens at the time of challenge from the body weight at the end of the experiments. Lesion score of the chickens from each group was investigated according to the method of Johnson and Reid [27] and oocyst value was calculated using the formula described by Peek and Landman [30], [31]. According to Merck Sharp & Dohme Company Ltd [32], ACI value of ≥180 was considered high performance, ACI value of 160-179 was considered as effective, and ACI value of <160 was considered as ineffective.

### Determination of serum antibody level and cytokine concentration

#### Preparation of the serum

Two-week-old chickens were randomly divided into three groups of 10 chickens each. The chickens in groups were respectively immunized with 200 µg recombinant protein of EaMIC5 without any adjuvant, pET-32a protein and the same PBS volume. One week later, a booster immunization was given with the same amount of components as the first immunization. After 10 days of booster immunization, the blood sample was collected, incubated at 37°C for 1 h and centrifugated at 5000 rpm for 5 min at 4°C to isolate the serum. The sera were used for the detection of antibody, cytokines and serum CD4, CD8.

#### Determination of serum antibody level by enzyme-linked immunosorbent assay (ELISA)

The IgG antibody levels against EaMIC5 in the serum samples were determined by ELISA as described previously [33]. Briefly, flat-bottomed 96-well plates (Marxi-Sorp, Nunc, Denmark) were coated overnight at 4°C with 100 µl solution per well of recombinant EaMIC5 (50 µg/ml) in 0.05 M carbonate buffer, pH 9.6. The plates were washed with 0.01 M PBS containing 0.05% Tween-20 (PBS-T) and blocked with 5% skim milk powder (SMP) in PBS-T for 2 h at 37°C. The plates were incubated for 2 h at 37°C with 100 µl of the serum samples diluted 1∶50 in PBS-T with 1% SMP in duplicate. After three washes, the plates were incubated for 1 h at room temperature with 100 µl/well of horseradish peroxidase-conjugated donkey anti-chicken IgG anti-body (Sigma) diluted 1∶1000 in 2% SMP in PBS-T. Color developmentwas carried out with 3,3′,5,5′-tetramethylbenzidine (TMB) (Sigma), and the optical density at 450 nm (OD_450_) was determined with a microplate spectrophotometer. All serum samples were investigated by ELISA at the same time under the same conditions and were included on one plate.

#### Determination of serum CD4, CD8 and cytokine concentrations

The concentration of soluble cluster of differentiation 4 (sCD4), soluble cluster of differentiation 8 (sCD8), interferon-γ (IFN-γ), interleukin-4 (IL-4), interleukin-10 (IL-10), interleukin-17 (IL-17), and tumor growth factor-β (TGF-β) in serum were detected by utilizing an indirect ELISA with the “chick cytokine ELISA Quantitation Kits” (catalog numbers: CSB-E13114C, CSB-E14317C, CSB-E08550Ch, CSB-E06756Ch, CSB-E12835C, CSB-E0467Ch, and CSB-E09875Ch for sCD4, sCD8, IFN-γ, IL-4, IL-10, IL-17 and TGF-β respectively; CUSABIO, China) in duplicate, according to manufacturer's instructions.

### Statistical analysis

One way analysis of variance (ANOVA) with Duncan's multiple range tests were used for the determination of statistical significance by using the SPSS statistical package (SPSS for Windows 16, SPSS Inc., Chicago, IL, USA) after all data in this paper was test by Levene (F)-Test by using SPSS. Differences among groups were tested and p<0.05 was considered to indicate a significant difference.

## Results

### Cloning and sequence analysis of EaMIC5

**Figure 1:**
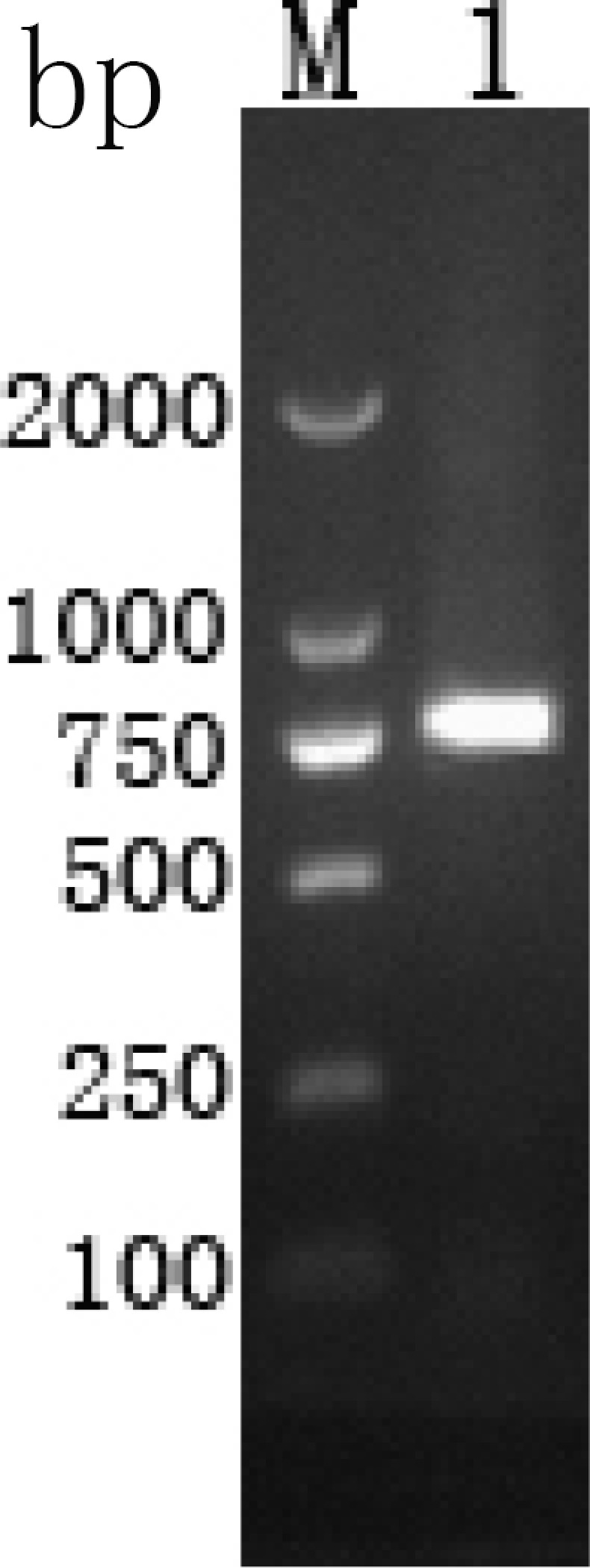
The ORF of EaMIC5 PCR. (Lane M) DNA Mark (ordinate values in bp); (Lane 1) the ORF of EaMIC5.

By overlapping the 3′- and 5′-RACE fragments, a transcript of 877 bp was obtained, which have been submitted to NCBI (GenBank Accession No. KF922373). A 178 bp 5′-untranslated region (5′-UTR) was detected before the ATG initiation codon and a 336 bp open reading frame (ORF) (Fig.1) was found that terminated with the TAA stop codon. On the 3′-end, the cDNA had a 363 bp 3′-UTR in the frame. By analysis with the sequence, the ORF was found to encode a protein of 111 amino acids with a molecular mass of 12.18 kDa. The theoretical pI of the protein was 9.44. No signal peptide, GPI anchor and transmembrane domain were found in the deduced protein, but one O-glycosylation site and twelve phosphorylation sites could be detected. The protein had six hydrophilicity regions including 7—17, 24—35, 38—56, 59—76, 82—90 and 96—111, and four high antigenic index and consecutive regions including 9—23, 25—57, 69—77 and 83—111. It also contained two Apple Factor XI like-regions.

### EaMIC5 multiple sequence alignment and cladogram

**Figure 2:**
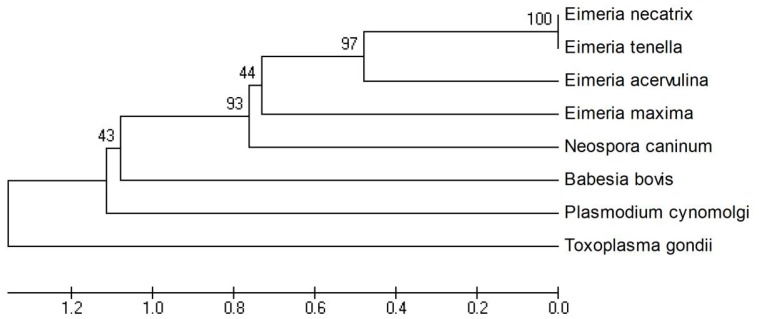
The phylogenetic tree of amino acid sequences between EaMIC5 and those of MIC5 from other species.

When compared with the known microneme nucleotide and protein sequences on the NCBI database (http://www.blast.ncbi.nlm.nih.gov/blast.cgi/) and on the GeneDB database (http://www.genedb.org/blast/submitblast/GeneDB_Eacervulina), the identity of EaMIC5 nucleotide sequence was 99% to *E. acervulina* hypothetical protein (EAH_00034850.1) and 60% to *E. acervulina* hypothetical protein (EAH_00013920.1) in GeneDB, and was lower than 15% to any other nucleotide sequence in NCBI. The amino acid sequence of EaMIC5 had 100% homology to the C-terminus of *E. acervulina* hypothetical protein (CDI78535.1) in NCBI. It also had 89% identity to *E. acervulina* hypothetical protein (EAH_00034850.1) in GeneDB, 42% identity to *E. necatrix* microneme protein 5 (ABY53156.1) and 40% identity to *E. tenella* microneme protein 5 (CAB52368.1) in NCBI. The phylogenetic tree of amino acid sequences was built by using MEGA4.0 and the result of cladogram (Fig.2) showed that the kinship of EaMIC5 protein between *Eimeria* spp was highly related when compared with other species (*Neospora caninum*, *Babesia bovis*, *Plasmodium cynomolgi*, *Toxoplasma gondii*) of apicomplexan parasites.

### Expression, purification and renaturation of recombinant EaMIC5

**Figure 3:**
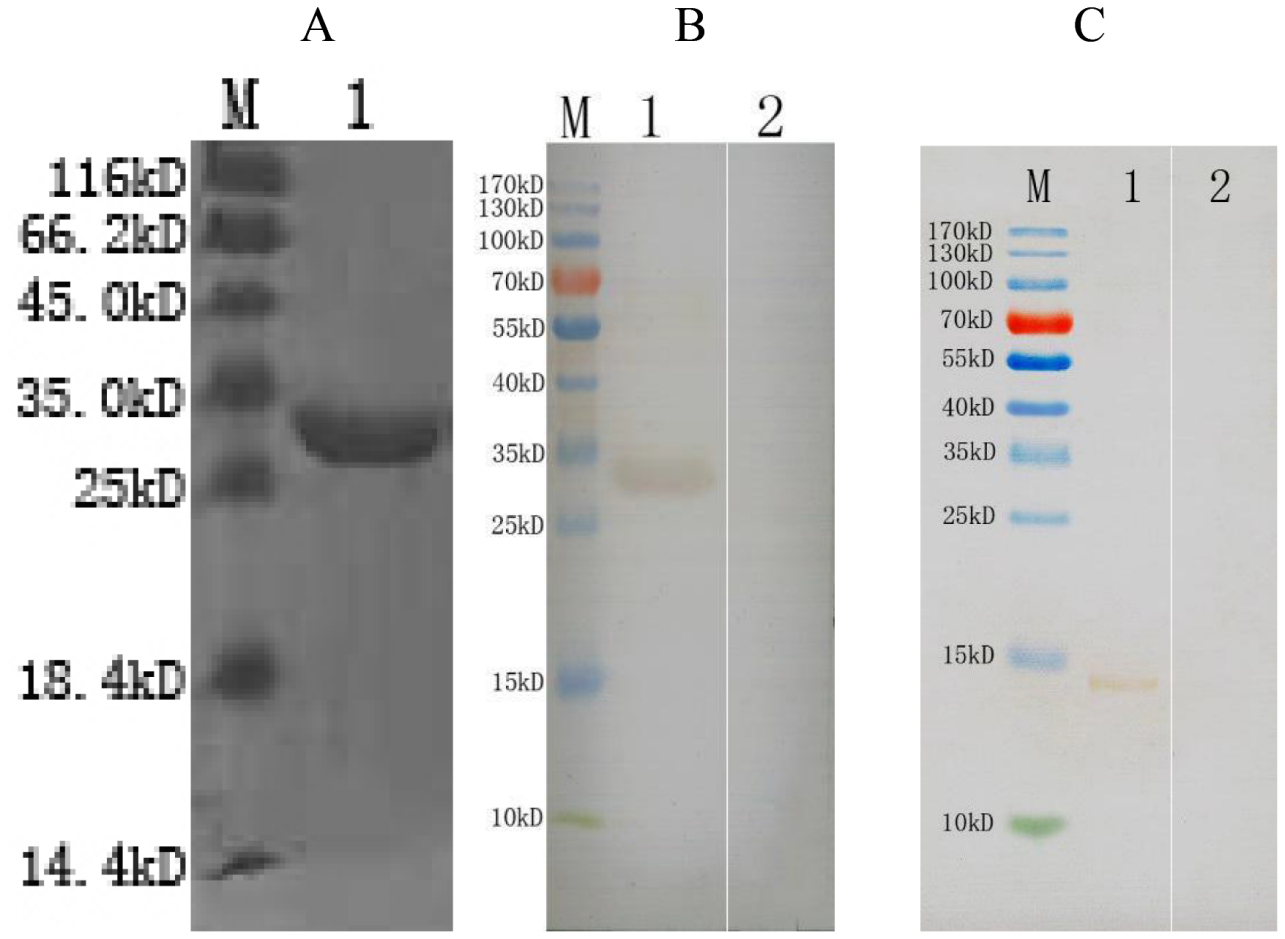
A: SDS-PAGE of recombinant protein purified. (Lane M) protein Mark (ordinate values in kDa); (Lane 1) MIC5 protein; B: Immunoblot for the recombinant EaMIC5. (Lane M) protein Mark (ordinate values in kDa); (Lane 1) Recombinant protein MIC5 probed by serum from chickens experimentally infected with *E. acervulina* as primary antibody; (Lane 2) Recombinant protein MIC5 probed by serum of normal chickens as primary antibody; C: Immunoblot for crude somatic extracts of *E. acervulina* sporozoites. (Lane M) protein Mark (ordinate values in kDa); (Lane 1) crude somatic extracts of *E. acervulina* sporozoites probed by serum from rat immunized by EaMIC5. (Lane 2) crude somatic extracts of *E. acervulina* sporozoites probed by serum of normal rat without immunizing as primary antibody.

SDS–PAGE showed that the recombinant protein was mostly found in the sonicated bacteria inclusion bodies. After purification from the inclusion bodies by chromatography on the Ni-NTA and renaturation, the protein was seen as a single band with the molecular mass 30 kDa on the SDS–PAGE gel (Fig.3A). Subtracted the 18 kDa fused protein in the vector, the recombinant protein molecular weight was consistent with the deduced size of 12.18 kDa.

### Immunoblot for the recombinant and native protein of EaMIC5

The results of the immunoblot assay indicated that the recombinant EaMIC5 protein was recognized by immune sera of chickens infected with *E. acervulina*, but could not be recognized by the serum of normal chickens (Fig.3B). Western blot analysis also showed that rat anti-EaMIC5 antiserum recognized the native EaMIC5 as a band of about 14 kDa in the somatic extract of *E. acervulina* sporozoites (Fig.3C), slightly larger than the deduced one.

### Expressions of EaMIC5 in sporozoites and merozoites

**Figure 4:**
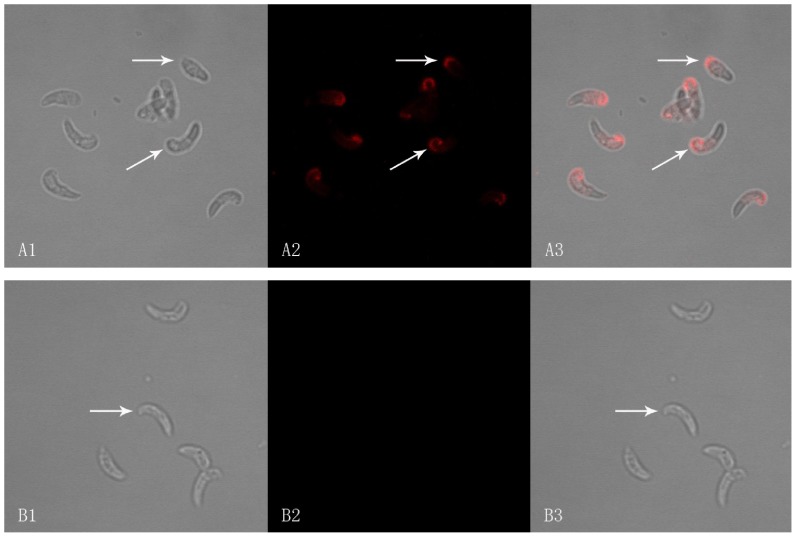
Expression of EaMIC5 protein in *E. acervulina* sporozoites by immunofluorescence assay (×100 magnification). Panel A: *E. acervulina* sporozoites. The white arrows indicate sporozoites. (A1) Differential interference contrast (DIC). (A2) Immunofluorescence localization using Cy3. (A3) DIC and Immunofluorescence combined. Panel B:Negative control, the sporozoites were probed by serum of normal rat without immunizing as primary antibody. (B1) DIC. (B2) Cy3. (B3) Combined.

Using anti-rEaMIC5 serum, EaMIC5 protein expression was investigated in sporozoites (Fig.4) and merozoites. The results showed that greater staining intensity was seen in sporozoites but in case of the merozoites the staining intensity was weak. No staining was seen in the negative control sections.

### Protective effects of vaccination on *E. acervulina* challenge

The immunization efficacies of EaMIC5 are described in Table 2. No chicken died from coccidial challenge in any group in this study. Body weight gains were significantly reduced in challenged control group, PBS control group and pET-32a protein control group compared with unchallenged control group (p<0.05). Chickens immunized with recombinant EaMIC5 protein displayed significantly enhanced weight gains relative to chickens in challenged control group, PBS control group and pET-32a protein control group (p<0.05). The oocyst counts of EaMIC5 immunized chickens were significantly lower than that of challenged control group, PBS control group and pET-32a protein control group (p<0.05). Significant alleviations in duodenal lesions were observed in EaMIC5 immunized chickens compared to that of challenged control group, PBS control group and pET-32a protein control group (p<0.05). Group of chickens immunized with recombinant protein resulted in ACI more than 160.

### IgG titers and concentrations of cytokines, CD4 and CD8 in sera of immunized chickens

**Figure 5:**
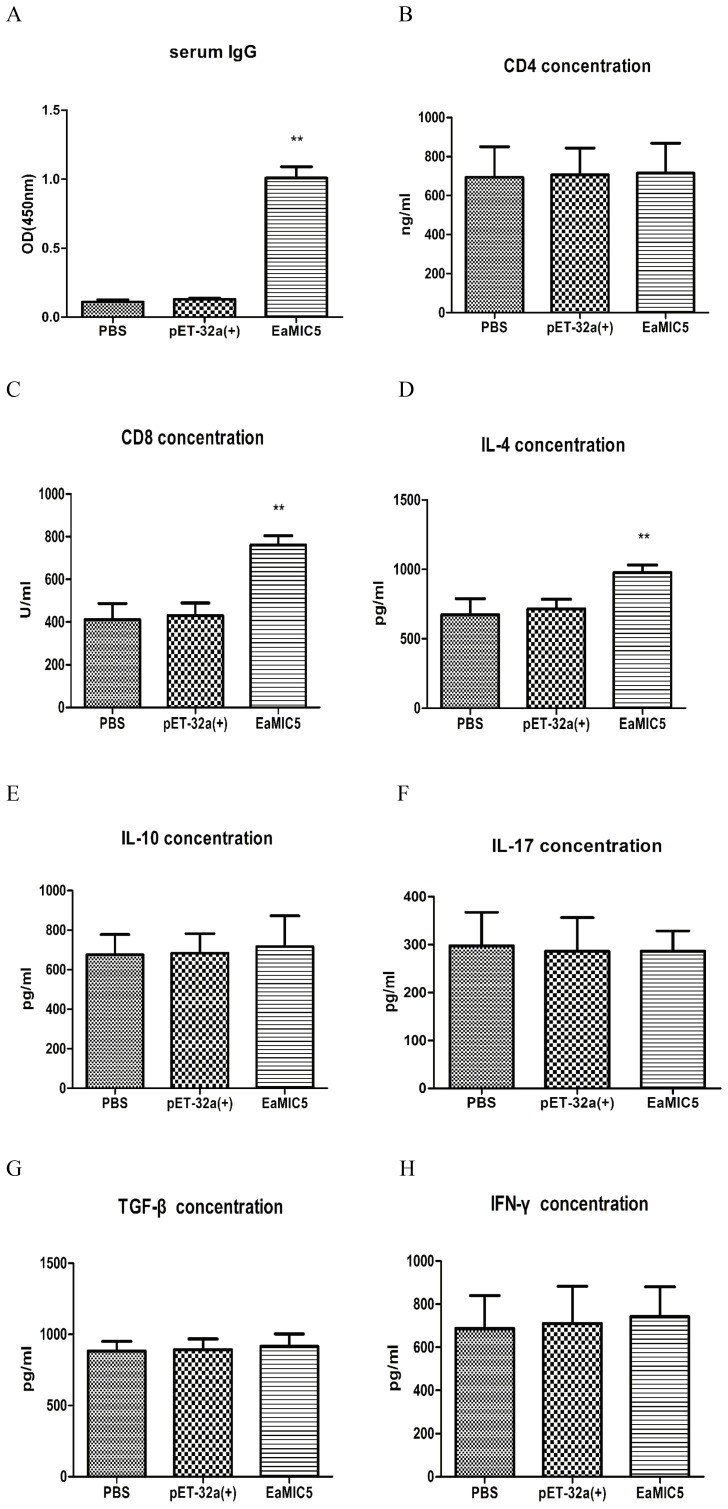
Serum EaMIC5-specific IgG, the concentrations of CD4, CD8 and cytokine levels in chickens. Chickens were immunized intramuscularly with PBS (negative control), pET-32a protein (pET-32a control), recombinant EaMIC5 protein. The IgG titers and the concentration CD4, CD8 and cytokine are expressed as mean ± SD with respect to absorbance at 450 nm. One star on the bar is slightly different from others, and two stars are significantly different (p<0.05), otherwise no different (p>0.05). A: IgG; B: CD4; C: CD8. D: IL-4; E: IL-10; F: IL-17; G: TGF- β and H: IFN-γ.

As depicted in Fig.5A, serum from chickens immunized with recombinant EaMIC5 protein showed significantly high level of IgG antibody (p<0.05) compared to that of controls, whereas IgG antibody of chicken immunized with pET-32a protein and PBS was not significantly induced (p>0.05). Meanwhile significantly higher levels of sCD8 (Fig.5C) and IL-4 (Fig.5D) were observed in chickens immunized with recombinant EaMIC5 protein compared to the control groups (p<0.05). There were no significant differences of the sCD4 and other cytokines between the immunized and control groups.

## Discussion

So far, complete sequences of MIC5 of *E. tenella* [17], *E. necatrix* and *E. maxima* [34] have been published in NCBI, but none of *E. acervulina* has been reported. In this research, we successfully obtained the complete sequence of MIC5 of *E. acervulina* and some characters of the protein were clarified.

In our current research, a nucleic acid sequence of 877 bp was obtained by RACE using the primers based on the EST of EaMIC5 in GenBank. This sequence contained a 336 bp open reading frame (ORF). The molecular mass of the deduced translation product of the ORF was about 12.18 kDa. The ORF included the start codon at the site 179—181 and the stop codon at the site 512—514. Compared with the sequences of MIC 5 of other *Eimeria* spp, the product was found to have 42% identity to *E. necatrix* MIC 5 (ABY53156.1) and 40% identity to *E. tenella* MIC 5 (CAB52368.1). The phylogenetic analysis of amino acid sequences indicated that this protein was highly related to the MIC 5 of *Eimeria* when compared with that of *Neospora caninum*, *Babesia bovis*, *Plasmodium cynomolgi*, *Toxoplasma gondii*. Compared to the EtMIC5 sequences published [17], we found that there were no transmembrane region and signal region for GPI anchor attachment in EaMIC5 sequences. BLASTP analysis revealed two Apple Factor XI like regions in the EaMIC5, which were similar to the eleven cysteine-rich receptor-like regions with striking similarity to the Apple domains (A-domains) of the binding regions of blood coagulation factor XI (FXI) [15] and plasma pre-kallikrein (PK) [16] in EtMIC5. According to Brown et al [17] and Saouros et al [18], EaMIC5, similar to EtMIC5 and TgMIC5, was located mainly at the apical tip of the sporozoite. In the current research, immunofluorescence analysis using serum against the recombinant protein showed that the native protein of EaMIC5 also mainly located at the apical end of the sporozoite. All of the above results suggested that the gene we obtained should be the MIC5 of *E. acervulina*. However, we found that TgMIC5, EtMIC5, EmMIC5 and EnMIC5 were larger than EaMIC5 in size and, compared to TgMIC5, the MIC5 proteins of *Eimeria* had no leader peptide. This indicated that MIC5s of different species might be different in size.

We found that EaMIC5 also had 100% homology to the C-terminus of *E. acervulina* hypothetical protein (CDI78535.1). However, the relationship between EaMIC5 and this hypothetical protein and whether the MIC5 was truncated from the same gene to the hypothetical protein in *E. acervulina* need to be further researched.

In this study, the sera against recombinant EaMIC5 recognized a band of about 14 kDa in the somatic extract of *E. acervulina* sporozoites. It indicated that the native EaMIC5 was 14 kDa in size. Comparison of the native EaMIC5 with the deduced one suggested that the native EaMIC5 was slightly larger than the deduced one of 12.18 kDa. It might be resulted from post-translational modification, such as glycosylation and phosphorylation. The sequence analysis indicated that there were glycosylation and phosphorylation sites in EaMIC5.

In this study, western blot assay revealed that recombinant EaMIC5 could be detected by the sera of chicken experimentally infected with *E. acervulina*, It indicated that EaMIC5 could be recognized by host immune system and induce the antibody response.

Identification of genes expressed in the life cycle of coccidian is very critical to understand the developmental biology of these parasites and MIC proteins including MIC5 are generally considered to be involved in cellular invasion. In this research, we demonstrated that MIC5 could be detected in sporozoite and merozoite stages of the life cycle. However, in sporozoite and merozoite the location of EaMIC5 differs. EaMIC5 is located mainly at the apical tip of the sporozoite and is similar to EtMIC2 in *E. acervulina* sporozoite [35], while in the merozoite EaMIC5 is diffused and located mainly at both poles. The reason for this difference maybe because the protein is confined to an intracellular location in resting sporozoites but is translocated to the parasite surface when it comes in contact with the cells [17].

This result proclaimed that the protein was conserved in the life cycle of this parasite, but the expression of this protein in other stages of *E. acervulina* and its detailed localization are worthy of further researches.

IL-4 is known to be a marker cytokine for Th2 cells, and it can promote B cell generation, differentiation, maturation, CD4^+^ cells differentiation to Th2 cells and the production of antibody [36]. In this study, EaMIC5 was found to be able to induce significant release of IL-4 and high levels of IgG antibody, indicating that EaMIC5 could enhance humoral response.

CD4^+^ and CD8^+^ cells have been shown to release a soluble forms known as sCD4 and sCD8 [37] and the sCD8 has long been used as a marker for the identification of active cytotoxic and suppressor cells [38]. The levels of sCD4 and sCD8 in serum are consistent with the count of CD4^+^ and CD8^+^ cell [39]. In this study, the concentration of sCD8 was significantly increased, suggesting that EaMIC5 was able to stimulate the recruitment of this T cell subpopulation. IFN-γ is the marker cytokine of Th1 type and the CD8^+^ cells are also well known for their ability to simultaneously produce high levels of IFN-γ in response to the parasite [40]. In this research, we attempted to investigate the involvement of IFN-γ in the immune response during immunization with EaMIC5. However, no significant IFN-γ was detected. The inconsistency of sCD8 with IFN-γ need to be further researched. In this study, it was also found that the concentrations of sCD4 did not differ significantly between EaMIC5 group and control groups. This also needs to be further investigated.

Zhu et al [9], [41] reported that antigen cSZ-JN1 could induce significant level of IL-4 and TGF-β, and cSZ-JN2 could stimulate significant level of IL-2 10 days post second injection. They also found neither cSZ-JN1 nor cSZ-JN2 could incite significant level of the IFN-γ at the same time point. In our study, we measured the level of cytokines at 10 days post booster injection and found that EaMIC5 could induce significant level of IL-4, but not incite significant level of the IFN-γ at the time point. These were consistent with Zhu's results. In addition, our results showed that EaMIC5 could not resulted in significant level of IL-10, IL-17 and TGF-β. Song et al [8] indicated none of pVAX-LDH, pVAX-LDH-IFN-γ and pVAX-LDH-IL-2 could induce the increment of CD4^+^ and CD8^+^ at 7 days post primary and booster immunization. But we found EaMIC5 could incite significant level of sCD8 while the level sCD4 could not be promoted 10 days post booster. Moreover, Qi et al [42] found that EtMIC1 induced significant level of the IFN-γ transcript profiles at first immunization (at days 14), and reached the peak at third immunization (at days 28). All of the above results indicated that different antigens might induce different cytokine and CD4^+^ or CD8^+^ cells at different time points. Thus, the profiles of cytokines and T cells stimulated by EaMIC5 at different time points should be further investigated.

In our research, recombinant EaMIC5 resulted in an ACI more than 160 in the animal protective experiment, suggesting that EaMIC5 might be an effective vaccine candidate against *E. acervulina*. Recent reports demonstrated that DNA vaccine or co-delivery of cytokines as adjuvants could enhance the efficiency of vaccines to induce strong humoral and cellular immune responses [43], [44]. So the possibilities of the DNA vaccine of EaMIC5 or co-delivery with cytokines and other molecular adjuvants to enhance the immunity of EaMIC5 is worthy of further researches.

In recent studies, dual immunofluorescence staining of EtMIC3 and EtMIC5 on fixed and permeabilized sporozoites of *E. tenella* and the parasites apically attached to host cells showed that EtMIC3 located mainly at the apical tip of the sporozoite, whilst the majority of EtMIC5 labeling was detected just posterior to this region [45]. EtMIC3 could bind to sialic acid-bearing molecules on the epithelial cell surface of the host, and played key roles in the invasion of the sporozoite [45], [46]. However, it was reported that the eleven cysteine-rich receptor-like regions similar to the Apple domains of EtMIC5 was associated with the parasites membrane and provided a crucial link between cytoskeletal elements of the parasite and receptors on the surface of the host cell [47]. TgMIC5 was also retained on the parasite plasma membrane via its physical interaction with the membrane-anchored toxoplasma subtilisin 1 (TgSUB1) and directly regulated MPP2 activity or influenced MPP2's ability to access substrate cleavage sites on the parasite surface [48]. In our current research, we demonstrated that EaMIC5 was located mainly at the apical tip of the sporozoite and could be translocated to the merozoite surface, and EaMIC5 could induce partial protection against the challenge of *E. acervulina*. All of these results suggested that EaMIC5 might also be involved in the cell invasion of *E. acervulina*. However, the exact role of EaMIC5 during cell invasion is still unclear and need further investigation.

In conclusion, we successfully obtained EaMIC5 full sequence based on the EST published on GenBank by the method of RACE. This protein was expressed in sporozoite and merozoite stages of the coccidian and could stimulate humoral and cellular responses to induce partial protection against the challenge of *E. acervulina*. Its location on sporozoite and merozoite and its protective function also indicated that EaMIC5 might be involved in the cell invasion. However, the exact roles of EaMIC5 in cell invasion need to be further studied.
